# Prediction of metastasis risk (11 year follow-up) using VEGF-R1, VEGF-R2, Tie-2/Tek and CD105 expression in breast cancer (*n*=905)

**DOI:** 10.1038/sj.bjc.6601452

**Published:** 2004-02-24

**Authors:** J P Dales, S Garcia, S Carpentier, L Andrac, O Ramuz, M N Lavaut, C Allasia, P Bonnier, C Taranger-Charpin

**Affiliations:** 1 Department of Pathology, Centre Hospitalier et Universitaire Nord, Marseille Cedex 20, France; 2 Department of Gynecologic Oncology, Hôpital de La Conception, Marseille, France

**Keywords:** VEGF-R1, VEGF-R2, Tie-2/Tek, CD105, breast cancer, prognosis

## Abstract

Neoangiogenesis in tumours contributes to the development of blood-borne metastases, and can be evaluated by markers of activated endothelial cells in preference to panendothelial markers. Our purpose was to document the prognostic significance of VEGF-R1, VEGF-R2, Tie-2/Tek and CD105 immunoexpression in breast carcinoma frozen samples (*n*=905, follow-up=11.7 years). We observed that: (i) CD105 (*P*=0.001) and Tie-2/Tek (*P*=0.025) (but not VEGF-R1 and VEGF-R2) overexpression correlated with a shorter survival, and were (Cox's model) independent histoprognostic indicators; (ii) only CD105 marked expression correlated (*P*=0.035) with a shorter survival of node-negative patients; (iii) three markers – CD105 (*P*=0.001), Tie-2/Tek (*P*=0.01), VEGF-R1 (*P*=0.001), but not VEGF-R2 – correlated with metastatic risk in node-negative patients in univariate analysis; and (iv) VEGF-R1 (*P*=0.01) expression correlated with high local recurrence risk. It is concluded that CD105 and to a lesser extent Tie-2/Tek and VEGF-R1, but not VEGF-R2 are endowed with prognostic significance that may be useful for patient monitoring, particularly CD105 expression for selecting node-negative patients for more aggressive postsurgery therapy.

Tumour growth and metastases require neoangiogenesis in tumour stroma. Neoangiogenesis is a complex multistep process including endothelial cell migration and proliferation, microvessel differentiation and anastomosis, and extracellular matrix remodelling (Folkman, 1995; Ellis and Fidler, 1996; Fox *et al*, 2001).

Quantification of neangiogenesis in many human solid tumours has been reported to be a prognostic marker (Weidner *et al*, 1992; Gasparini and Harris, 1995; Toi *et al*, 1996; Vermeulen *et al*, 1996), particularly in breast carcinomas where extensive neovascularisation proved to be an indicator of poor prognosis (Horak *et al*, 1992).

Intratumoural microvessel density was previously evaluated using panendothelial markers such as CD34, CD31 and von Willebrand factor (Gasparini, 1996a; Fox *et al*, 2001). However, these markers may not be specific of neoangiogenesis and may not stain all tumour blood vessels to the same degree, and results in terms of prognostic significance are controversial (Gasparini, 1996a,1996b; Vermeulen *et al*, 1996; Charpin *et al*, 1997). More recently, markers of activated endothelial cells, such as CD105 (Seon *et al*, 1997; Kumar *et al*, 1999; Fonsatti *et al*, 2001), Tie-2/Tek (Brown *et al*, 1995; Mustonen and Alitalo, 1995; Takahashi *et al*, 1995; Yokoyama *et al*, 2000) and VEGF (Salven *et al*, 1996; Peters *et al*, 1998) receptors as prognostic indicators more suitable for identifying stromal vessels resulting from tumour neoangiogenesis, have been reported. However, in breast carcinomas, the prognostic significance of these markers has not been evaluated in a large series and long-term follow-up.

Our purpose in this study was to document the variations of VEGF-R1, VEGF-R2, Tie-2/Tek and CD105 expression in a large series of breast carcinomas (*n*=905), and to correlate the immunohistochemical expression on frozen sections of these markers with patient outcome (11.7 years follow-up) in terms of overall survival and metastasis- and recurrence-free survival. We specifically tried to determine whether these markers could be of clinical relevance for selecting node-negative patients who could benefit, after the initial surgery, from more aggressive therapy. We also tried to determine whether these markers were equivalent or not, and to rank them for better cost-effectiveness in routine use.

## MATERIALS AND METHODS

### Patients

The study included 905 patients aged 25–81 years (mean±s.d.=56±13, 1 years) with breast carcinoma who underwent surgery from January 1986 to December 1994. They did not receive chemotherapy or hormone therapy before surgery. Axillary node excision (*n*=802) combined with wide local excision with margin clearance of mastectomy was realised in the Department of Oncologic Gynecology (*Hôpital de La Conception*). All specimens were examined by the same group of three senior pathologists experienced in breast carcinoma diagnosis and screening (CC, LA, SG).

The follow-up period ranged from 6 to 15 years (median 11.7 years). The records for 2001 showed that 338 patients (36.8%) relapsed, among whom 228 died (median survival: 82 months) and 567 (63.2%) were disease free. Overall survival was calculated as the period from surgery until date of death. Metastasis- and recurrence-free survival were calculated as the period from surgery until the date of first metastasis and recurrence.

The mean tumour size was 20.5±13.7 mm; 24% of tumours were 10 mm or smaller, 42% between 10 and 20 mm, 19% were more than 20 mm but less than 30 mm and 15% were larger than 30 mm.

Histologic examination of surgical specimens was performed on paraffin-embedded sections stained with haematoxylin, eosin and safranin.

Tumours were classified as ductal carcinomas (67%), lobular carcinomas (19%) and as carcinomas of other types, including tubular, mucinous, medullary, papillary, apocrine and mixed (14%). They were distributed as tumour grade 1 (23%), grade 2 (52%) and grade 3 (25%). Tumour grading, initially assessed using Scarff, Bloom and Richardson scores (Bloom and Richardson, 1957), was re-evaluated according to Elston and Ellis (1991) and Ellis and Fidler (1996).

A mean of 14.4 (s.d.±4.1) lymph nodes was found in axillary node excision, and 449 patients (56%) were node negative.

### Immunostaining procedure and quantification of VEGF-R1, VEGF-R2, Tie-2/Tek and CD105 immunostained vessels

Fresh tissue fragments were sampled by pathologists (CC, LA, SG) immediately after intraoperative diagnosis as described previously (Charpin *et al*, 1988,^1995^).

Detection of Tie-2/Tek expression and CD105 was carried out with a polyclonal mouse anti-human Tie-2 (C-20) and anti-CD105 (E2) (Novocastra Tebu, Le Perray en Yvelines, 78610 France). The detection of VEGF-R1 (FLT-3) and VEGF-R2 (KDR) was carried out with mouse monoclonal antibodies (Sigma-Aldrich, 38297 St Quentin Fallavior, France). Automated immunoperoxidase procedures were performed using the Ventana Gene II device with Ventana kits (Ventana, Strasbourg, France).

The detection of antigenic sites of oestrogen and progesterone receptors was performed as reported previously (Charpin *et al*, 1988).

The microvessel count was assessed in the most vascularised areas (hotspots) (Partanen *et al*, 1992; Maisonpierre *et al*, 1993; Mustonen and Alitalo, 1995; Takahashi *et al*, 1995; Folkman and D'Amore, 1996) using a × 20 objective (1.060 mm^2^ field diameter) with a Zeiss Axioplan microscope (Carl Zeiss International; Gottingen, Germany). The mean value of the vessel count in the four fields was retained as the final value.

With anti-Tie-2/Tek and anti-VEGF-R1 and -R2 antibodies, the vessel labelling was incomplete and only some endothelial cells were focally stained. Therefore, Tie-2/Tek- and VEGF-R1 and -R2- positive staining could not be evaluated in terms of vessel count, but only in terms of positive stromal surface semiquantitatively assessed.

### Statistical analysis

The Kaplan–Meier method was used to analyse disease-free and overall survival rates. The difference between curves was evaluated with the Mantel Cox test (or log-rank test) for observations regarding censored survival or events. All computations were carried out with NCSS 2000 statistical software (Ness, Kaysville, UT, USA) as reported previously (Charpin *et al*, 1997), and the cutoff value was validated with *P*-value curves (Altman *et al*, 1994).

## RESULTS

### Tie-2/Tek, VEGFR-1, VEGF-R2 and CD105 distribution in tissue sections

Tie-2/Tek, VEGF-R1 and VEGF-R2 distribution was heterogeneous and was observed as focally visible linear deposits in some endothelial cells along vessel walls. The immunostained surface was small, no more than 20% (mean=11.58%, s.d.±4.87, median 12%; mean=8.4%, s.d.=4.03, median=7%; mean=10.7%, s.d.=4.6, median=11%, respectively).

CD105 were observed in most endothelial cells along the cell membrane and/or within the cytoplasm. The staining was strong, delineating the vessel outlines and permitted the vessel count. The mean number of CD105-positive vessels was 13.9 (s.d.±6.4) (Zeiss, Axiophot, × 20 objective).

### Univariate (Kaplan–Meier and log-rank) analysis and Tie-2/Tek, VEGF-R1, VEGF-R2 and CD105 prognostic significance

#### VEGF-R1/VEGF-R2

VEGF-R1 and VEGF-R2 expressions did not significantly correlate with the patients' overall survival. However, greater VEGF-R1 expression (cutoff 5%), but not of VEGF-R2, correlated with a higher risk of metastases (*P*=0.03), in particular for node-negative patients (*P*=0.001) (Table 1

**Table 1:** VEGF-R1 immunoexpression (cutoff=5% stained surface) and metastasis-free survival in node-negative patients

	**VEGF-R1 <5% (n=128)**	**VEGF-R1 ⩾5 %(n=321)**
Metastasis−	30	86
Metastasis+	98	235

, Figure 1

**Figure 1:**
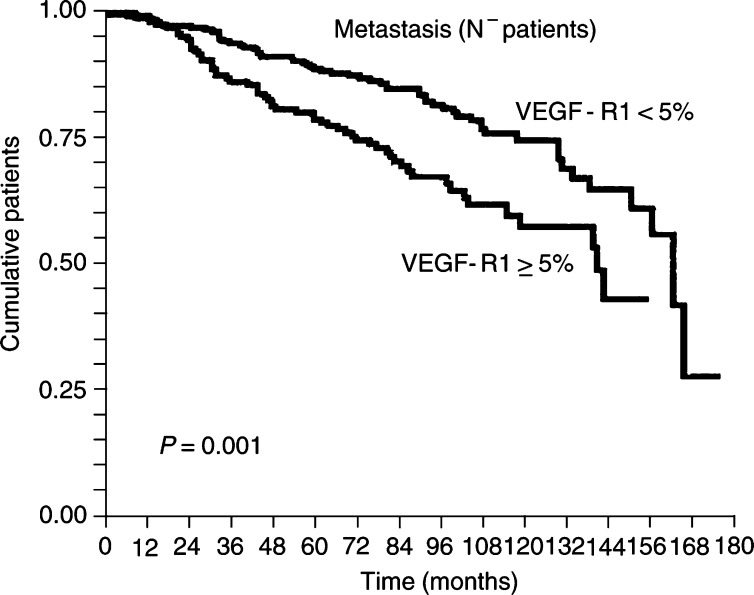
Kaplan–Meier survivorship plot of metastasis-free survival: a greater risk of metastasis was found (*P*=0.03, not shown) for patients with tumours in which VEGF-R1-stained surface was greater (cutoff 5%), and more specifically in node-negative patients (*p*=0.001).

). Also, higher expression (cutoff 5%) of VEGF-R1, but not of VEGF-R2, correlated (*P*=0.01) with a higher risk of relapse, particularly in node-negative patients (Figure 2

**Figure 2:**
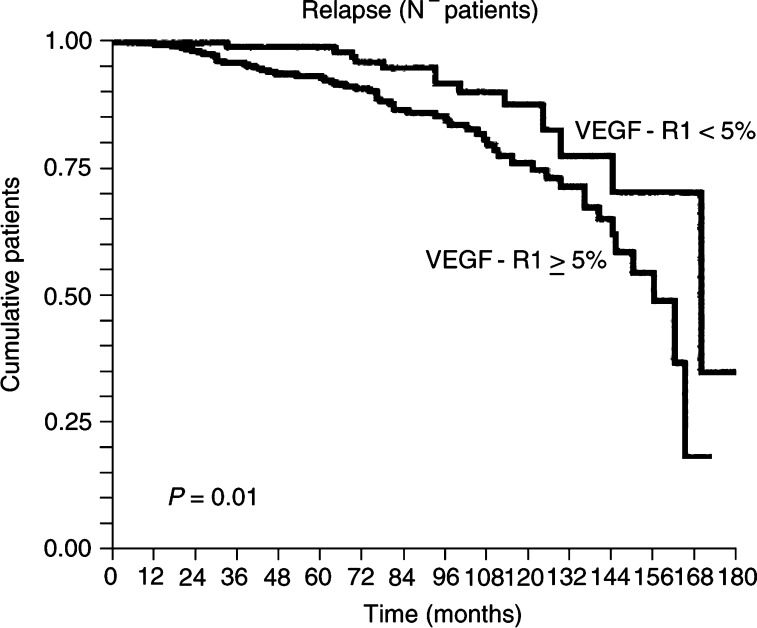
Kaplan–Meier survivorship plot of recurrence-free survival: a greater risk of recurrence was found in which VEGF-R1-stained surface within tumours was greater in node-negative patients (*P*=0.01).

, Table 2

**Table 2:** VEGF-R1 immunoexpression (cutoff 5% of stained surface) and recurrence-free survival in node-negative patients

	**VEGF-R1 <5% (n=128)**	**VEGF-R1 ⩾5% (n=321)**
Recurrence−	13	55
Recurrence+	115	266

), and shorter disease-free survival (*P*=0.007). The evaluation of optimal cutoff was determined with *P* curves (Altman *et al*, 1994).

#### ^*^Tie-2/Tek

**Figure 3:**
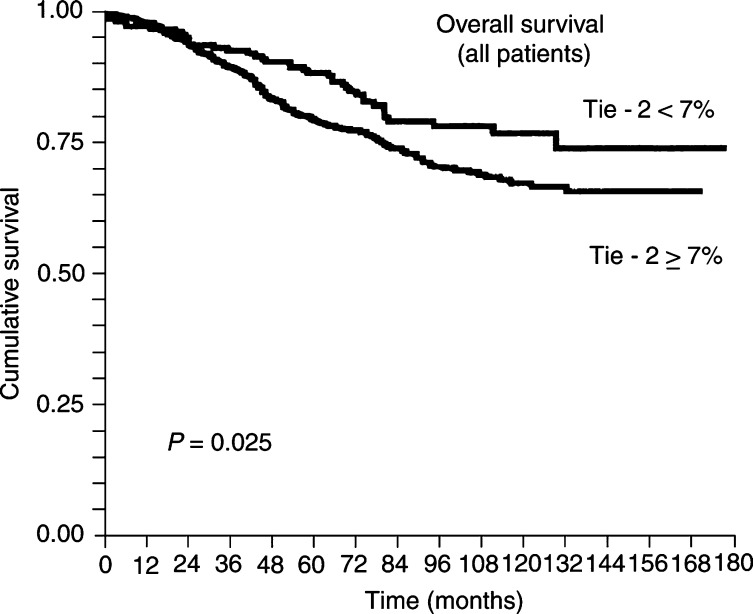
Kaplan–Meier survivorship plot of overall patients (but not for node-negative patients) with breast carcinomas: a greater risk of death was found for patients with tumours in which 7% or more of the tumour stained surface was Tie-2/Tek positive (*P*=0.025).

The Tie-2 immunostained tumour tissue surface (cutoff point=7%) correlated (*P*=0.025) with overall survival (Figure 3

, Table 3

**Table 3:** Tie-2/Tek immunoexpression (cutoff=7% of stained surface) and overall survival

	**Tie-2 <7% (n=200)**	**Tie-2 ⩾7% (n=705)**
Deceased	39	184
Alive	161	521

). Tumours with a greater (⩾7%) Tie-2-positive surface were associated with poorer survival as compared to those that exhibited a smaller Tie-2/Tek-positive surface. The evaluation of optimal cutoff was also determined with the *P* curve according to the recommendations of Altman *et al* (1994). However, when node-negative patients were evaluated, Tie-2 immunoexpression did not exhibit prognostic significance (*P*>0.05).

**Figure 4:**
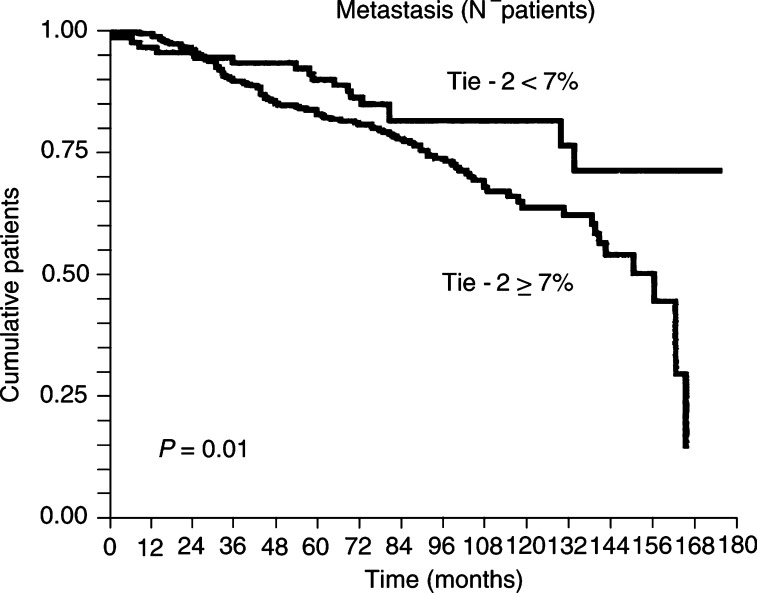
Kaplan–Meier univariate analysis showing a higher risk of metastasis in node-negative patient with tumours in which Tie-2/Tek stained surface was larger than 7% (*P*=0.01).

In contrast, Tie-2 immunostained surface correlated with a greater risk of early metastasis in node-negative patients (*P*=0.01) (Figure 4

, Table 4

**Table 4:** Tie2/Tek immunoexpression (cutoff=7% of stained surface) and metastasis-free survival in node-negative patients

	**Tie-2 <7% (n=333)**	**Tie-2 ⩾7% (n=116)**
Metastasis+	80	36
Metastasis−	253	80

) and with shorter disease-free survival (local recurrence+metastases) (*P*=0.003).

#### ^*^CD105

**Figure 5:**
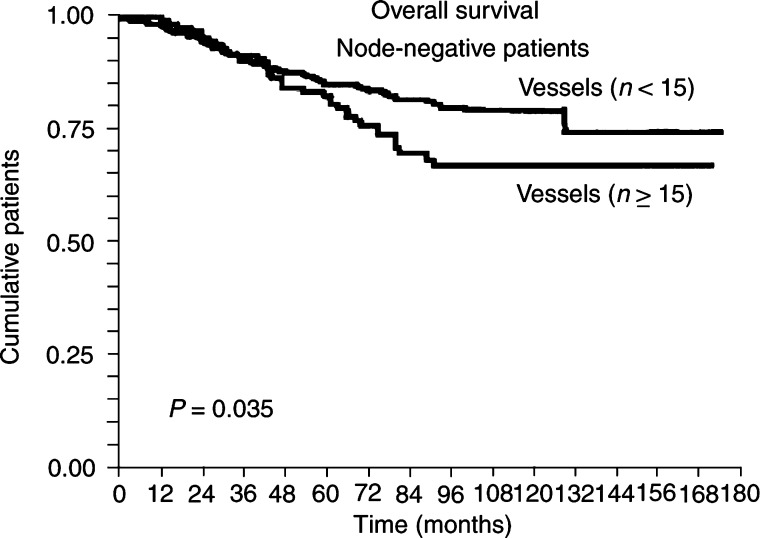
Kaplan–Meier univariate analysis showing a higher risk of death in node-negative patients (*P*=0.035) with a number of CD105 positive ⩾15 within tumours.

The number of CD105-positive vessels (cutoff=15) correlated (*P*=0.001) with overall survival, particularly in the node-negative subset of patients (*P*=0.035) as shown in Figure 5

and Table 5

**Table 5:** CD105 immunoexpression (cutoff of number of vessels=15) and survival in node-negative patients

	**CD105 <15 (n=311)**	**CD105 ⩾15 (n=138)**
Deceased	57	37
Alive	254	101

**Figure 6:**
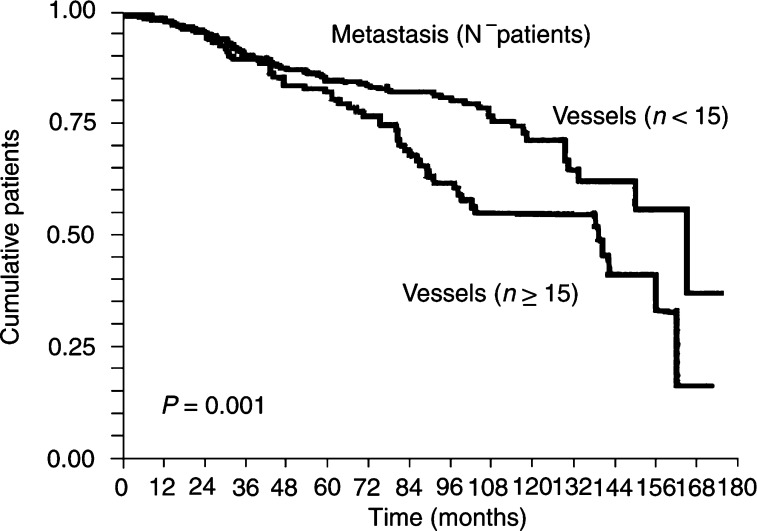
Kaplan–Meier univariate analysis showing a higher risk of metastasis in node-negative patients (*P*=0.001) with a number of CD105 positive ⩾15 within tumours.

. Similarly, the number of CD105-positive vessels (>15) correlated significantly (*P*=0.0002) with a higher risk of metastases in all patients and in the subset of node-negative patients (*P*=0.001) (Figure 6

).

### Multivariate (Cox's model) and prognostic significance

In multivariate analysis, a high degree of Tie-2/Tek and CD105 expression, but not of VEGF-R1-positive staining, proved to be a prognostic indicator independent of other known histoprognostic factors such as tumour size and grade, histological type and hormone receptors, in terms of survival and metastasis risk (Table 6

**Table 6:** Multivariate analysis (Cox's/log-rank)

	**Probability level overall survival (all patients)**	**Probability level overall survival (N− patients)**	**Probability level metastasis-free survival (N− patients)**
VEGF-R1 (cutoff=5)	*P*=0.4540	*P*=0.07631	*P*=0.3812
CD105 (cutoff=15)	*P*=0.038	*P*=0.0041	*P*=0.0366
Tie-2/Tek (cutoff=7)	*P*=0.0273	*P*=0.0924	*P*=0.01808
Tumour grade	*P*=0.0084	*P*=0.0029	*P*=0.0394
ER (>20%)	*P*=0.0224	*P*=0.0781	*P*=0.1774
PR (>20%)	*P*=0.0721	*P*=0.0161	*P*=0.1861
Histological type	*P*=0.4251	*P*=0.2298	*P*=0.4552
Tumour size	*P*=0.3752	*P*=0.3871	*P*=0.6916

^N−^ patients=node-negative patients; VEGF-R1=Vascular endothelial growth factor receptor 1; ER=Oestrogen receptors; PR=projesteron receptors.

).

## DISCUSSION

Although several experimental studies have suggested that molecules expressed by activated endothelial cells tend to produce new vessels in human malignant tumours including breast cancer, only one clinicopathological study (Kumar *et al*, 1999) has documented the prognostic significance of these potential markers, which could allow the selection of those node-negative patients with early breast cancer who might benefit from more aggressive therapy and eventually from specific antiangiogenic therapy (Lin *et al*, 1997,^1998^; Matsuno *et al*, 1999; Siemeister *et al*, 1999). However, although several previous reports have shown the potential clinical relevance of these markers, whose expression correlates with the extent of tumour vasculature and aggressiveness, the immunohistochemical expressions of Tie-2/Tek [Kaipainen *et al*, 1994; Hatva *et al*, 1995; Salven *et al*, 1996; Peters *et al*, 1998) and of VEGF-R1 and VEGF-R2 (Yokoyama *et al*, 2000; Yao *et al*, 2001) expression have not been well documented in the literature, with regard to their clinicopathological relevance.

Our results show that in univariate analysis (Kaplan–Meier), greater immunocytochemical expression of CD105 (*P*=0.001) and of Tie-2/Tek (*P*=0.025) significantly correlates with a poor overall survival, but this is not the case for VEGF-R1 and -R2. In addition, CD105 (*P*=0.035), but not Tie-2/Tek, retained a prognostic significance in terms of overall survival in node-negative patients. Furthermore, multivariate analysis (Cox's/log rank) showed that CD105 and Tie-2/Tek were independent of other current prognostic indicators (tumour size and grade, histological type, oestrogen and progesterone receptors). No previous reports have shown that CD105 correlated with overall survival of node-negative patients, but this result corroborates the study of Kumar *et al* (1996) in which marked CD105 immunoexpression correlated with overall 5-year survival of all patients. Our results show that, in terms of overall survival, CD105 expression in breast carcinomas is a stronger prognostic indicator as compared to Tie-2/Tek and that VEGF-R1 and -R2 have no prognostic value.

We observed that greater expression of CD105 (*P*=0.0002), Tie-2/Tek (*P*=0.0067) and VEGF-R1 (*P*=0.03), but not of VEGF-R2, correlated with greater metastatic risk. Similar correlations were observed for node-negative patients with the three markers – CD105 (*P*=0.001), Tie-2/Tek (*P*=0.01) and VEGF-R1 (*P*=0.001). Moreover, in multivariate analysis, all three were independent indicators of metastatic risk. No previous report has documented VEGF-R1, Tie-2/Tek and CD105 prognostic value in breast carcinomas in terms of this risk.

Furthermore, we observed that Tie-2/Tek (*P*=0.003) and VEGF-R1 (*P*=0.01), but not VEGF-R2 and CD105, correlated with early local recurrence, and VEGF-R1 (*P*=0.01) correlated with early local recurrence in node-negative patients. This result shows that Tie-2/Tek and VEGF-R1 are better indicators of prognosis in terms of local relapse, in contrast to CD105 that is a better indicator in terms of overall survival. Thus, the three markers are of similar value in terms of metastatic risk, whereas VEGF-R2 has no prognostic value.

The method used to evaluate microvessel density in tumours has been diversely evaluated, likely explaining the conflicting results in the literature (see review in Gasparini, 1996b, Charpin *et al*, 1997) when antipanendothelial CD34, CD31 or von Willebrand factor antibodies were applied in the immunocytochemical procedure. In the present study, we investigated CD105, Tie-2/Tek and VEGF-R1 and -R2, on frozen sections (Leica 3050) with automated immunodetection (Ventana Gene II), which constitutes optimal conditions for antigen preservation and for procedure standardisation. Our goal was firstly to determine in these optimal conditions, the real respective value of these markers in terms of prognosis in node-negative patients and secondly to further develop the procedure on paraffin sections that is considered more easy to perform routinely (work in preparation). Anti-CD105 antibodies stained the complete vessel outlines, most endothelial cells expressing a high level of CD105. In contrast, anti-Tie-2/Tek and likewise anti-VEGF-R1 and anti-VEGF-R2 antibodies stained some cells or part of endothelial cells in a given vessel. Therefore, the evaluation of CD105 as the number of vessels in ‘hotspots’ (Weidner *et al*, 1991, Weidner, 1995) was possible, but Tie-2/Tek, VEGF-R1 and -R2 had to be evaluated as percentage of stained stromal surface.

Our study shows that CD105 immunohistochemical expression on frozen sections in breast carcinomas is an independent prognostic indicator, better than Tie-2/Tek in terms of overall survival for node-negative patients. CD105, Tie-2/Tek, VEGF-R1, but not VEGF-R2 expression, also correlate with high metastatic risk in node-negative patients. Only Tie-2/Tek and VEGF-R1 expression correlate with early local recurrence in node-negative patients. Therefore, immunodetection of markers specific of activated endothelial cells can be considered as potentially useful for patient monitoring, more specifically for node-negative patients with a poorer prognosis who might benefit from more aggressive therapy and further antigenic therapy.
